# Supplementary material for: Nanotechnology in action: silver nanoparticles for improved eco-friendly remediation
Source: PeerJ. 2024 Oct 3;12:e18191. doi: 10.7717/peerj.18191 (PMC11456292; doi:10.7717/peerj.18191)
Supplement: Supplemental Information 1 [file peerj-12-18191-s001.docx]

**Table S1** Examination of relative prices, including a rundown of about how much each kind of nanoparticle costs per mass.

**Nanoparticle Cost per Gram* Cost per Kilogram***

Silver (AgNPs) 100 - 500 100,000 - 500,000

Titanium Dioxide (TiO_2_) 0.01 - 0.05 10 - 50

Iron Oxide (Fe_3_O_4_) 0.05 - 0.1 50 - 100

Zinc Oxide (ZnO) 0.02 - 0.1 20 - 100

* Amount on USA dollars
