# Supplementary material for: Nanotechnology in action: silver nanoparticles for improved eco-friendly remediation
Source: PeerJ. 2024 Oct 3;12:e18191. doi: 10.7717/peerj.18191 (PMC11456292; doi:10.7717/peerj.18191)
Supplement: Supplemental Information 2 [file peerj-12-18191-s002.docx]

**Table S2.** Biological systems used for AgNP synthesis (2022-2024)

| **Organism** | **Size and shape of AgNPs** | **Whole cell or extract used** | **Approach (biosorption or bioreduction)** | **References** |
| --- | --- | --- | --- | --- |
| **Bacteria** |  |  |  |  |
| *Streptococcus pneumoniae* | 7.37±4.55 nm, spherical, | Aqueous filtrate containing the bacterial metabolites | Bioreduction | (Mishra et al. 2024) |
| *Magnetospirillum*  *magnetotacticum* | Width: 2–42 nm, length: 32–59 nm, rod-shaped | Whole cell | Biosorption | (Sancho et al. 2023) |
| *Cyanobacterium Cyanothece sp.* | 70-140 nm, average diameter 84.37 nm, spherical | Whole cell | Biosorption | (Mohamed et al. 2022) |
| *Aggregatimonas sangjinii gen. nov.* | 5.5-4.5 nm, spherical and polygonal | Whole cells | Biosorption | (Chung et al. 2022) |
|  |  |  |  |  |
| **Fungus** |  |  |  |  |
| *Embellisia spp. and Gymnoascus spp* | 2–20 nm, spherical | Aqueous filtrate containing the fungal metabolites | Bioreduction | (Mohammed et al. 2024) |
| *Emericella dentata* | 10-80 nm | Aqueous filtrate containing the fungal metabolites | Bioreduction | (Alqaraleh et al. 2023) |
| *Beauveria bassiana and Metarhizium anisopliae* | 23-101 nm | Aqueous filtrate containing the fungal metabolites | Bioreduction | (Soleimani et al. 2022) |
| *Aspergillus sp. and Alternaria sp* | 4.5 ± 20 - 50.2 ± 74 nm, spherical | Aqueous filtrate containing the fungal metabolites | Bioreduction | (Mostafa et al. 2022) |
| *Fusarium oxysporum* | 100 nm | Aqueous filtrate containing the fungal metabolites | Bioreduction | (Allend et al. 2022) |
|  |  |  |  |  |
| **Algae** |  |  |  |  |
| *Spirulina platensis* | 2.23-14.68 nm, spherical | Aqueous filtrate containing the algal compounds | Bioreduction | (Doman et al. 2024) |
| *Desmodesmus abundans* | 14.9 ± 6.4 - 18.3 ± 7.5 nm, and  127.8 ± 14.8 nm | Aqueous filtrate containing the algal compounds | Bioreduction | (Mora-Godinez et al. 2023) |
|  |  |  |  |  |
| **Yeast** |  |  |  |  |
| *Saccharomyces cerevisiae* | 5-30 nm, spherical to oval | Cell free yeast extract | Bioreduction | (Kim et al. 2024) |
|  |  |  |  |  |
| **Plant** |  |  |  |  |
| *Ocimum kilimandscharicum* | methanolic extract: 69.72 ± 0.45 nm; aqueous extract: 70.14 ± 1.15 nm | Methanolic and aqueous leaf extracts | Bioreduction | (Ouandaogo et al. 2024) |
| *Pongamia pinnata* | ~ 116 nm, spherical | Seed extracts | Bioreduction | (Telange et al. 2024) |
| *Dillenia indica* | 50.17 nm | Flower extract | Bioreduction | (Gupta et al. 2024) |
| *Ocimum gratissimum* (gr-AgNPs) *O. tenuiflorum* (te-AgNPs) *and O. americanum* (am-AgNPs) | 69.0 +/- 5 nm for te-AgNPs,  46.9 +/- 9 nm for gr-AgNPs,  58.5 +/- 18.7 nm for am-AgNPs | Leaf extract | Bioreduction | (Alex et al. 2024) |
| *Aconitum Leave* | 5-10 nm, spherical | Crude extract of plant material | Bioreduction | (Ahmad et al. 2024b) |
| *Vigna unguiculata (L) Walp* | 5 to 13 nm | Leaf extract | Bioreduction | (Mulu et al. 2024) |
| *Citrus sinensis* | 10-50 nm | Peel extract | Bioreduction | (Hani et al. 2024) |
| *Alpinia galanga* | 20-25 nm, spherical | Methanolic extract of rhizome | Bioreduction | (Ahmad et al. 2024a) |
| *Mentha piperita, Mentha spicata and Mentha longifolia* | 30-63nm, cuboidal | Methanolic extract | Bioreduction | (Aftab et al. 2024) |
| *Moringa oleifera* | 24 to 40 nm | Leaf extract | Bioreduction | (Shaaban et al. 2023) |
| *Galium aparine* (G-AgNPs) *and Helichrysum arenarium* (H-AgNPs) | 52.0+/-10.9 nm (G-AgNPs),  23.9+/-1.0 nm (H-AgNPs) | Aqueous extract | Bioreduction | (Ozdemir et al. 2023) |
| *Cullen tomentosum* | 45 nm |  | Bioreduction | (Asong et al. 2023) |
| *Ziziphus spina-christi* | 11.25 nm, spherical | Aqueous leaf extract | Bioreduction | (Abdelaziz et al. 2023) |
| Saw palmetto seed | 11.17-38.32 nm, spherical | Phenolic compounds were extracted | Bioreduction | (Abdel-Aty et al. 2023) |
| Fruit waste: grape seed extracts | ~ 20 nm | Seed extract | Bioreduction | (Zhao et al. 2022) |
| *Garcinia mangostana* | 2.36 ± 0.87–3.74 ± 1.11 nm, and  189.96 ± 71.27–294.73 ± 106.46 nm | Aqueous peel extract | Bioreduction | (Srikhao et al. 2022) |
| *Tilia sp.* | 4-46 nm | Plant extract | Bioreduction | (Simonova et al. 2022) |
| *Saccharum officinarum* | 10-50 nm. | Plant extract | Bioreduction | (Saruchi et al. 2022) |
| *Vitis vinifera* | 68.2 nm | Cane extract | Bioreduction | (Michailidu et al. 2022) |
| *Knoxia sumatrensis* | 7.73-32.84 nm, hexagona | Aqueous leaf extract | Bioreduction | (Loganathan et al. 2022) |
| *Alternanthera sessilis* | 15-40 nm, spherical | Aqueous leaf extract | Bioreduction | (Kabeerdass et al. 2022) |
| Pomegranate peel | 15-25 nm | Aqueous extract | Bioreduction | (Goda et al. 2022) |
| *Eugenia uniflora linnaeus* | 32 nm | Fruit extract | Bioreduction | (Franzolin et al. 2022) |
| *Curcuma longa* | 5-25 nm | Leaf extract | Bioreduction | (Fatimah et al. 2022) |
| *Coleus forskohlii* | 10-50 nm, trigonal, hexagonal, spherical, rod | Leaf extract | Bioreduction | (Chakraborty et al. 2022) |
| Commercially available green tea | 30-50 nm, spherical | Aqueous leaf extract | Bioreduction | (Ashikbayeva et al. 2022) |
| *Rumex vesicarius* | 27 nm | Aqueous leaf extract | Bioreduction | (Elsebaie et al. 2023) |

**References (Supplementary Table S2)**

Abdel-Aty AM, Barakat AZ, Bassuiny RI, and Mohamed SA. 2023. Statistical optimization, characterization, antioxidant and antibacterial properties of silver nanoparticle biosynthesized by saw palmetto seed phenolic extract. *Sci Rep* 13:15605. 10.1038/s41598-023-42675-0

Abdelaziz AM, Elshaer MA, Abd-Elraheem MA, Ali O, Haggag MI, El-Sayyad GS, and Attia MS. 2023. Ziziphus spina-christi extract-stabilized novel silver nanoparticle synthesis for combating Fusarium oxysporum-causing pepper wilt disease: in vitro and in vivo studies. *Arch Microbiol* 205:69. 10.1007/s00203-023-03400-7

Aftab R, Akbar F, Afroz A, Asif A, Khan MR, Rehman N, and Zeeshan N. 2024. Mentha piperita silver nanoparticle-loaded hydrocolloid film for enhanced diabetic wound healing in rats. *J Wound Care* 33:xlviii-lx. 10.12968/jowc.2024.33.Sup3a.xlviii

Ahmad E, Athar A, Nimisha, Zia Q, Sharma AK, Sajid M, Bharadwaj M, Ansari MA, and Saluja SS. 2024a. Harnessing nature's potential: Alpinia galanga methanolic extract mediated green synthesis of silver nanoparticle, characterization and evaluation of anti-neoplastic activity. *Bioprocess Biosyst Eng*. 10.1007/s00449-024-02993-7

Ahmad S, Xu Q, Tariq M, Song M, Liu C, and Yan H. 2024b. Assessing the Potential of Aconitum Laeve Extract for Biogenic Silver and Gold Nanoparticle Synthesis and Their Biological and Catalytic Applications. *Molecules* 29. 10.3390/molecules29112640

Alex AM, Subburaman S, Chauhan S, Ahuja V, Abdi G, and Tarighat MA. 2024. Green synthesis of silver nanoparticle prepared with Ocimum species and assessment of anticancer potential. *Sci Rep* 14:11707. 10.1038/s41598-024-61946-y

Allend SO, Garcia MO, da Cunha KF, de Albernaz DTF, da Silva ME, Ishikame RY, Panagio LA, Nakazaro G, Reis GF, Pereira DB, and Hartwig DD. 2022. Biogenic silver nanoparticle (Bio-AgNP) has an antibacterial effect against carbapenem-resistant Acinetobacter baumannii with synergism and additivity when combined with polymyxin B. *J Appl Microbiol* 132:1036-1047. 10.1111/jam.15297

Alqaraleh M, Khleifat KM, Abu Hajleh MN, Farah HS, and Ahmed KA. 2023. Fungal-Mediated Silver Nanoparticle and Biochar Synergy against Colorectal Cancer Cells and Pathogenic Bacteria. *Antibiotics (Basel)* 12. 10.3390/antibiotics12030597

Ashikbayeva Z, Aitkulov A, Atabaev TS, Blanc W, Inglezakis VJ, and Tosi D. 2022. Green-Synthesized Silver Nanoparticle-Assisted Radiofrequency Ablation for Improved Thermal Treatment Distribution. *Nanomaterials (Basel)* 12. 10.3390/nano12030426

Asong JA, Frimpong EK, Seepe HA, Katata-Seru L, Amoo SO, and Aremu AO. 2023. Green Synthesis of Characterized Silver Nanoparticle Using Cullen tomentosum and Assessment of Its Antibacterial Activity. *Antibiotics (Basel)* 12. 10.3390/antibiotics12020203

Chakraborty A, Haque SM, Ghosh D, Dey D, Mukherjee S, Maity DK, and Ghosh B. 2022. Silver nanoparticle synthesis and their potency against multidrug-resistant bacteria: a green approach from tissue-cultured Coleus forskohlii. *3 Biotech* 12:228. 10.1007/s13205-022-03295-z

Chung D, Jung J, Kim JYH, Kim KW, and Kwon YM. 2022. Aggregatimonas sangjinii gen. nov., sp. nov., a novel silver nanoparticle synthesizing bacterium belonging to the family Flavobacteriaceae. *Antonie Van Leeuwenhoek* 115:325-335. 10.1007/s10482-021-01700-w

Doman KM, Gharieb MM, Abd El-Monem AM, and Morsi HH. 2024. Synthesis of silver and copper nanoparticle using Spirulina platensis and evaluation of their anticancer activity. *Int J Environ Health Res* 34:661-673. 10.1080/09603123.2022.2163987

Elsebaie EM, El-Wakeil NHM, Khalil AMM, Bahnasy RM, Asker GA, El-Hassnin MF, Ibraheim SS, El-Farsy MFA, Faramawy AA, Essa RY, and Badr MR. 2023. Silver Nanoparticle Synthesis by Rumex vesicarius Extract and Its Applicability against Foodborne Pathogens. *Foods* 12. 10.3390/foods12091746

Fatimah I, Hidayat H, Purwiandono G, Khoirunisa K, Zahra HA, Audita R, and Sagadevan S. 2022. Green Synthesis of Antibacterial Nanocomposite of Silver Nanoparticle-Doped Hydroxyapatite Utilizing Curcuma longa Leaf Extract and Land Snail (Achatina fulica) Shell Waste. *J Funct Biomater* 13. 10.3390/jfb13020084

Franzolin MR, Courrol DDS, de Souza Barreto S, and Courrol LC. 2022. Eugenia uniflora L. Silver and Gold Nanoparticle Synthesis, Characterization, and Evaluation of the Photoreduction Process in Antimicrobial Activities. *Microorganisms* 10. 10.3390/microorganisms10050999

Goda RM, El-Baz AM, Khalaf EM, Alharbi NK, Elkhooly TA, and Shohayeb MM. 2022. Combating Bacterial Biofilm Formation in Urinary Catheter by Green Silver Nanoparticle. *Antibiotics (Basel)* 11. 10.3390/antibiotics11040495

Gupta A, Pandey BC, Verma J, Tiwari I, Sahu AN, Manhas RK, and Kumari N. 2024. Biosynthesis of silver nanoparticle from flower extract of Dillenia indica and its efficacy as antibacterial and antioxidant. *Microb Pathog* 193:106779. 10.1016/j.micpath.2024.106779

Hani U, Kidwan FN, Albarqi LA, Al-Qahtani SA, AlHadi RM, AlZaid HA, Haider N, and Ansari MA. 2024. Biogenic silver nanoparticle synthesis using orange peel extract and its multifaceted biomedical application. *Bioprocess Biosyst Eng*. 10.1007/s00449-024-03031-2

Kabeerdass N, Murugesan K, Arumugam N, Almansour AI, Kumar RS, Djearamane S, Kumaravel AK, Velmurugan P, Mohanavel V, Kumar SS, Vijayanand S, Padmanabhan P, Gulyas B, and Mathanmohun M. 2022. Biomedical and Textile Applications of Alternanthera sessilis Leaf Extract Mediated Synthesis of Colloidal Silver Nanoparticle. *Nanomaterials (Basel)* 12. 10.3390/nano12162759

Kim DY, Kim M, Sung JS, Koduru JR, Nile SH, Syed A, Bahkali AH, Seth CS, and Ghodake GS. 2024. Extracellular synthesis of silver nanoparticle using yeast extracts: antibacterial and seed priming applicationss. *Appl Microbiol Biotechnol* 108:150. 10.1007/s00253-023-12920-7

Loganathan S, Selvam K, Shivakumar MS, Senthil-Nathan S, Vasantha-Srinivasan P, Gnana Prakash D, Karthi S, Al-Misned F, Mahboob S, Abdel-Megeed A, Ghaith A, and Krutmuang P. 2022. Phytosynthesis of Silver Nanoparticle (AgNPs) Using Aqueous Leaf Extract of Knoxia sumatrensis (Retz.) DC. and Their Multi-Potent Biological Activity: An Eco-Friendly Approach. *Molecules* 27. 10.3390/molecules27227854

Michailidu J, Matatkova O, Kolouchova I, Masak J, and Cejkova A. 2022. Silver Nanoparticle Production Mediated by Vitis vinifera Cane Extract: Characterization and Antibacterial Activity Evaluation. *Plants (Basel)* 11. 10.3390/plants11030443

Mishra M, Ballal A, Rath D, and Rath A. 2024. Novel silver nanoparticle-antibiotic combinations as promising antibacterial and anti-biofilm candidates against multiple-antibiotic resistant ESKAPE microorganisms. *Colloids Surf B Biointerfaces* 236:113826. 10.1016/j.colsurfb.2024.113826

Mohamed ME, El Semary NA, and Younis NS. 2022. Silver Nanoparticle Production by the Cyanobacterium Cyanothece sp.: De Novo Manipulation of Nano-Biosynthesis by Phytohormones. *Life (Basel)* 12. 10.3390/life12020139

Mohammed AE, Korany SM, Sonbol H, Alhomaidi EA, Alwakeel SS, and Elbaz RM. 2024. Myco-fabricated silver nanoparticle by novel soil fungi from Saudi Arabian desert and antimicrobial mechanism. *Sci Rep* 14:15211. 10.1038/s41598-024-63117-5

Mora-Godinez S, Contreras-Torres FF, and Pacheco A. 2023. Characterization of Silver Nanoparticle Systems from Microalgae Acclimated to Different CO(2) Atmospheres. *ACS Omega* 8:21969-21982. 10.1021/acsomega.3c01914

Mostafa EM, Abdelgawad MA, Musa A, Alotaibi NH, Elkomy MH, Ghoneim MM, Badawy M, Taha MN, Hassan HM, and Hamed AA. 2022. Chitosan Silver and Gold Nanoparticle Formation Using Endophytic Fungi as Powerful Antimicrobial and Anti-Biofilm Potentialities. *Antibiotics (Basel)* 11. 10.3390/antibiotics11050668

Mulu M, Tefera M, Guadie A, and Basavaiah K. 2024. Biosynthesis, characterization and study of the application of silver nanoparticle for 4-nitrophenol reduction, and antimicrobial activities. *Biotechnol Rep (Amst)* 42:e00838. 10.1016/j.btre.2024.e00838

Ouandaogo HS, Diallo S, Odari E, and Kinyu J. 2024. Silver nanoparticle biosynthesis utilizing Ocimum kilimandscharicum leaf extract and assessment of its antibacterial activity against certain chosen bacteria. *PLoS One* 19:e0295463. 10.1371/journal.pone.0295463

Ozdemir C, Gencer M, Coksu I, Ozbek T, and Derman S. 2023. A new strategy to achieve high antimicrobial activity: green synthesised silver nanoparticle formulations with Galium aparine and Helichrysum arenarium. *Arh Hig Rada Toksikol* 74:90-98. 10.2478/aiht-2023-74-3684

Sancho RE, Govindsamy A, and Pillay K. 2023. Optimization of Growth Conditions for Magnetospirillum magnetotacticum and Green Synthesis of Metallic Nanoparticles. *Applied Sciences* 13:8491.

Saruchi, Kaur M, Kumar V, Ghfar AA, and Pandey S. 2022. A Green Approach for the Synthesis of Silver Nanoparticle-Embedded Chitosan Bionanocomposite as a Potential Device for the Sustained Release of the Itraconazole Drug and Its Antibacterial Characteristics. *Polymers (Basel)* 14. 10.3390/polym14091911

Shaaban MT, Zayed M, and Salama HS. 2023. Antibacterial Potential of Bacterial Cellulose Impregnated with Green Synthesized Silver Nanoparticle Against S. aureus and P. aeruginosa. *Curr Microbiol* 80:75. 10.1007/s00284-023-03182-7

Simonova Z, Krbeckova V, Vilamova Z, Dobrocka E, Klejdus B, Cieslar M, Svoboda L, Bednar J, Dvorsky R, and Seidlerova J. 2022. The Effects of Nature-Inspired Synthesis on Silver Nanoparticle Generation. *ACS Omega* 7:4850-4858. 10.1021/acsomega.1c05308

Soleimani P, Mehrvar A, Michaud JP, and Vaez N. 2022. Optimization of silver nanoparticle biosynthesis by entomopathogenic fungi and assays of their antimicrobial and antifungal properties. *J Invertebr Pathol* 190:107749. 10.1016/j.jip.2022.107749

Srikhao N, Ounkaew A, Srichiangsa N, Phanthanawiboon S, Boonmars T, Artchayasawat A, Theerakulpisut S, Okhawilai M, and Kasemsiri P. 2022. Green-synthesized silver nanoparticle coating on paper for antibacterial and antiviral applications. *Polym Bull (Berl)*:1-18. 10.1007/s00289-022-04530-6

Telange DR, Mahajan NM, Mandale T, More S, and Warokar A. 2024. Pongamia pinnata seed extract-mediated green synthesis of silver nanoparticle loaded nanogel for estimation of their antipsoriatic properties. *Bioprocess Biosyst Eng*. 10.1007/s00449-024-03058-5

Zhao X, Tian R, Zhou J, and Liu Y. 2022. Multifunctional chitosan/grape seed extract/silver nanoparticle composite for food packaging application. *Int J Biol Macromol* 207:152-160. 10.1016/j.ijbiomac.2022.02.180
