# Supplementary material for: Nanotechnology in action: silver nanoparticles for improved eco-friendly remediation
Source: PeerJ. 2024 Oct 3;12:e18191. doi: 10.7717/peerj.18191 (PMC11456292; doi:10.7717/peerj.18191)
Supplement: Supplemental Information 3 — Reprinted with permission from (Hidayat et al., 2022). Copyright (2022), Elsevier. [file peerj-12-18191-s003.pdf]

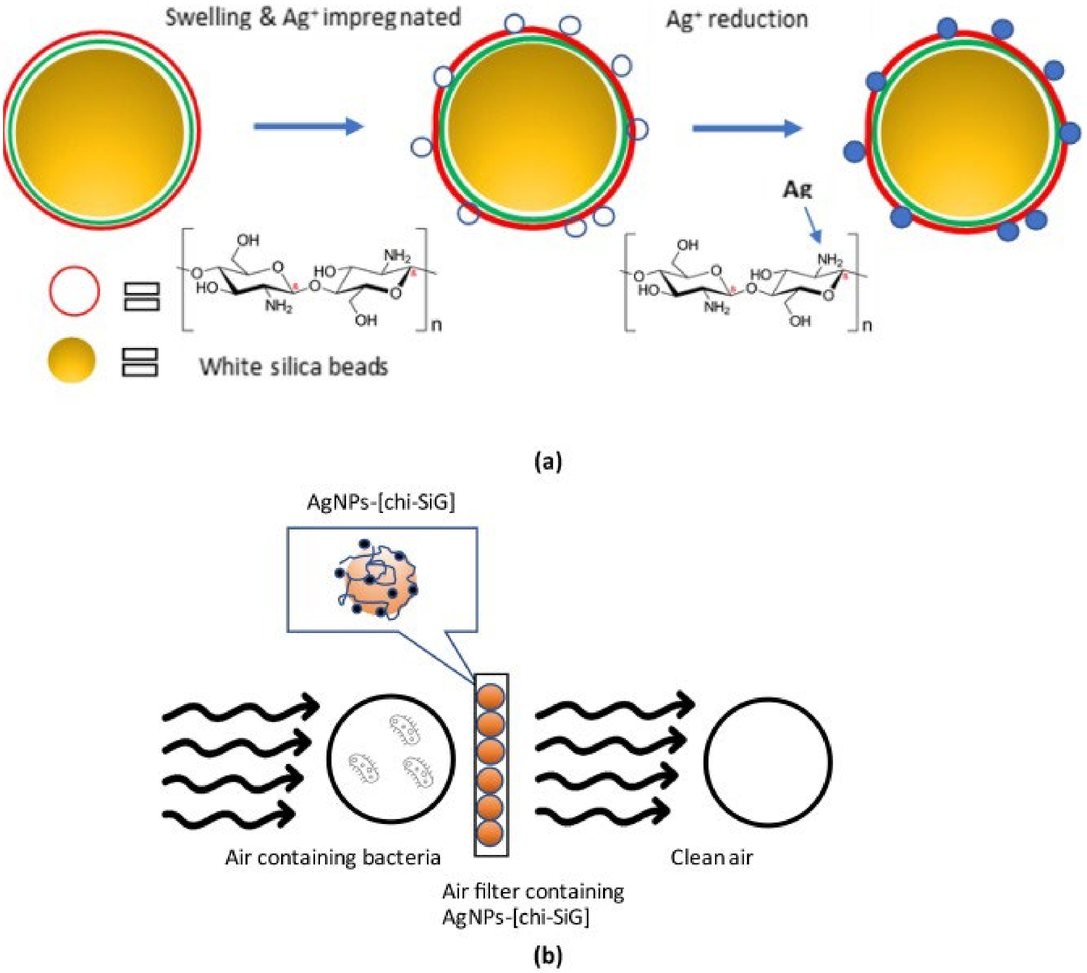

Figure 4. Preparation and antibacterial action of AgNPs. Reprinted with permission from (Hidayat et al. 2022). Copyright (2022), Elsevier
