# Supplementary figures and images for: Nanotechnology in action: silver nanoparticles for improved eco-friendly remediation
Source: PeerJ. 2024 Oct 3;12:e18191. doi: 10.7717/peerj.18191 (PMC11456292; doi:10.7717/peerj.18191)

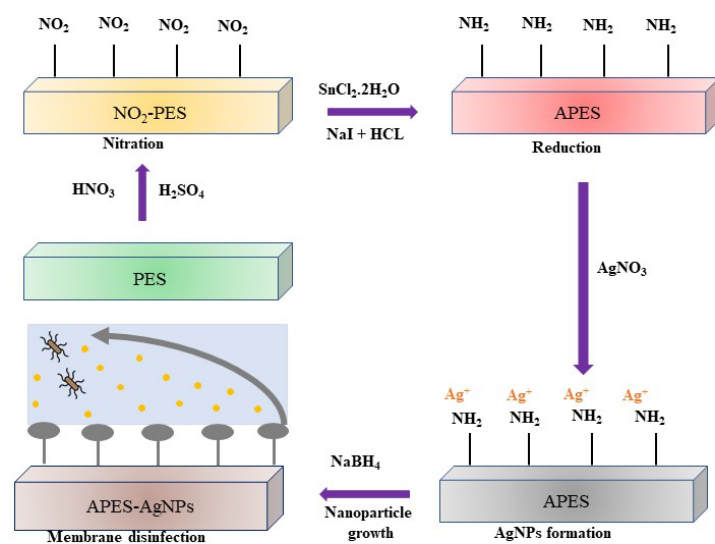

**Figure 5.** Preparation of aminated polyether sulfone decorated by AgNPs.

Supplement: Supplemental Information 4 [file peerj-12-18191-s004.pdf]

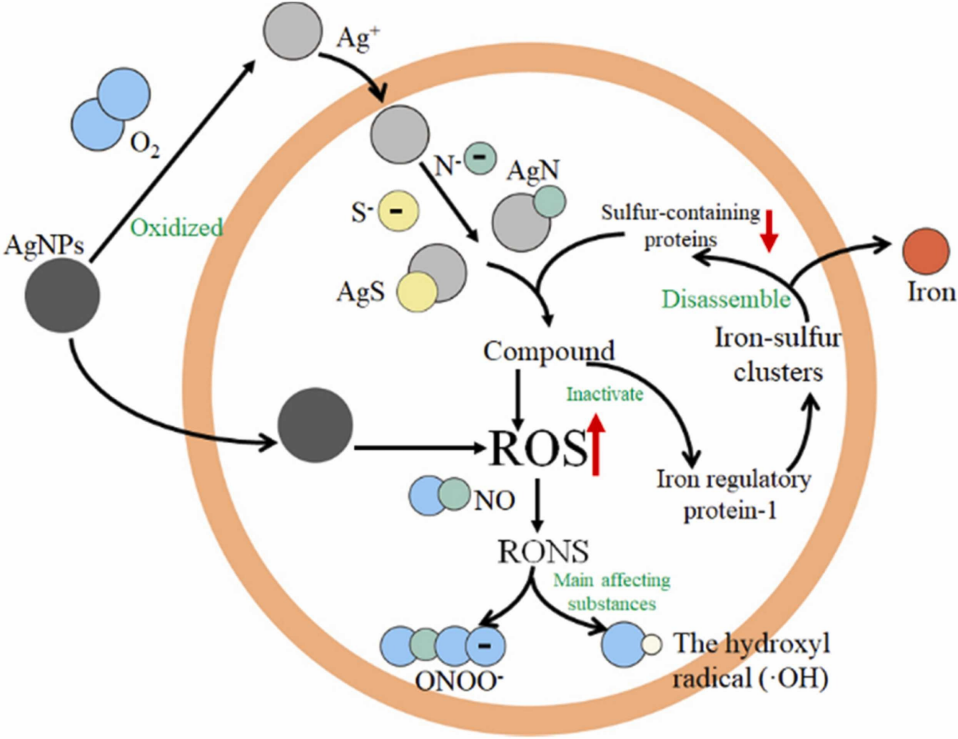

Supplement: Supplemental Information 5 — Reprinted with permission from (Nie et al., 2023). Copyright (2022), Elsevier [file peerj-12-18191-s005.pdf]
